# Whole Genome Data Uncover the Complex Origins of Polish Konik Horses

**DOI:** 10.3390/ani16111669

**Published:** 2026-05-29

**Authors:** Adrianna D. Musiał, Katarzyna Ropka-Molik, Tomasz Szmatoła, Przemysław Podstawski, Carrie J. Finno, Agnieszka Bieniek, Jarosław Wilczyński, Monika Stefaniuk-Szmukier

**Affiliations:** 1 Department of Animal Molecular Biology, National Research Institute of Animal Production, 32-083 Balice, Poland; tomasz.szmatola@iz.edu.pl (T.S.); przemyslaw.podstawski@iz.edu.pl (P.P.); agnieszka.bieniek@iz.edu.pl (A.B.); monika.stefaniuk@iz.edu.pl (M.S.-S.); 2 Faculty of Veterinary Medicine, University of Agriculture in Kraków, 30-059 Kraków, Poland; 3 Department of Population Health and Reproduction, School of Veterinary Medicine, University of California-Davis, Davis, CA 95616, USA; cjfinno@ucdavis.edu; 4 Institute of Systematics and Evolution of Animals, Polish Academy of Sciences, 31-016 Kraków, Poland; wilczynski@isez.pan.krakow.pl

**Keywords:** *Equus caballus*, WGS, SNPs, Polish Konik, genetic diversity, horse breeding, Tarpan

## Abstract

Polish Konik is a native horse breed from Poland often regarded as living descendants of the extinct Tarpan, which has been crucial to the breed’s conservation status and cultural importance. To evaluate Polish Konik origins, we sequenced 12 whole genomes and compared them with 148 horse genomes, including those of modern breeds, ancient Polish horses, Przewalski horses, and the Tarpan. The ADMIXTURE results showed that Polish Konik is dominated by a single ancestral component, which is also common among ancient Polish samples and several primitive breeds. The PCA places Polish Konik in a compact cluster positioned adjacent to the ancient and primitive samples. Both analyses indicate that Polish Konik share an ancestral component with several ancient and primitive-type horse samples. The NJ analysis indicates more complex origins of the breed and its close relationship with the Dülmener. None of the analyses showed a closer relationship of Polish Konik to the Tarpan than other analyzed breeds. The study demonstrates that while Polish Konik shares similarities with ancient and primitive horse populations, its genetic background is more complex, reflecting contributions from modern breeds. Our results indicate a close relationship with the Dülmener pony and do not provide sufficient evidence for the hypothesis of the direct origin of the Polish Konik from the extinct Tarpan.

## Simple Summary

For years, the origins of the Polish Konik horses remained unknown, but it was believed that the breed was a modern descendant of the extinct, wild Tarpan. We evaluated the breed’s origins by sequencing the complete genomes of 12 Polish Konik horses and comparing these sequences with those of other horses, both modern and ancient, using bioinformatic analysis. The results showed that Polish Konik shares similarities with ancient and primitive horse populations, but its genetic background is more complex, reflecting contributions from modern breeds. Therefore, we conclude that Polish Konik horses have a mixed heritage from various horse populations. This discovery does not provide sufficient evidence for the hypothesis of the Polish Konik’s direct origin from the Tarpan but could help guide the breeding and conservation of this special breed.

## 1. Introduction

Since World War II, the increasing specialization in horse breeding has led to the decline of non-profitable herds, resulting in a significant reduction in populations of breeds unsuitable for modern utility purposes. In response, international initiatives have been launched to protect locally adapted, small-population breeds as part of genetic resource conservation programs. The Polish Konik, a primitive-type horse, is among the Polish breeds included in such efforts. The Polish Konik is a hardy, native breed from Poland, often regarded as living direct descendants of the alleged Tarpan (*Equus ferus ferus*)—the elusive wild horse that once roamed the forests of Europe before vanishing in the 19th century [1]. The horse’s body is strong, with a stocky build and a dun coat coloration reminiscent of wild horses. They are managed in traditional studs as well as semi-ferally in reserves and are used for rewilding and habitat protection. Their primitive traits and cultural importance make Polish Konik an interesting subject of study.

The idea of the Polish Konik being a direct descendant of the Tarpan was strongly shaped in the last century by Professor Tadeusz Vetulani, a pioneering researcher who not only coined the name “Polish Konik” but also introduced it to the scientific literature and, beginning in the 1920s, dedicated his research to documenting and preserving what he believed to be the genetic and phenotypic legacy of the Tarpan within this breed. In 1928, Vetulani proposed a ground-breaking theory distinguishing a specific subspecies of the steppe Tarpan (*Equus gmelini*, as defined by Antonius in 1912), which he termed the forest Tarpan (*Equus gmelini sylvatica*). He envisioned the restitution of this forest form through selective breeding, using the remaining population of the Polish Konik as its living template. This idea was rooted in observed anatomical and morphological similarities, particularly in bone and skull structure, between the Polish Konik and known Tarpan specimens. Moreover, Vetulani noted a striking geographical overlap between the historical range of the last wild Tarpans and the native habitat of the Polish Konik. His breeding strategy aimed to preserve and emphasize primitive traits reminiscent of the forest Tarpan, selectively protecting individuals that best represented this ancestral type [2].

Unfortunately, World War II led to a sharp decline in the Polish Konik population in the Białowieża reserve. According to Vetulani [3], only 15 horses remained after the war, including five from the original herd and ten likely born during the conflict. This marked the beginning of the modern phase of Polish Konik breeding. Following Vetulani’s death in 1952, the Ministry of Agriculture closed the Białowieża reserve and established two new breeding centers. By 1954, guidelines for continued breeding had been defined. Following actions such as closing the studbooks and including the breed in genetic resource conservation programs, the population has been rebuilt to 2985 individuals as of 2025, descending from 16 dam and six sire founding lines (latest data for 2025, Polish Horse Breeders Association; www.pzhk.pl/hodowla/statystyka-hodowlana/ accessed on 28 April 2026).

Previous genetic studies on Polish Konik included analysis of genetic diversity [4,5], population structure [6], or the inbreeding coefficient [7], but did not mention the potential origin of the breed. Over the last decade, most research has included genetic analysis as Sanger sequencing of mitochondrial DNA [6,8] and microsatellite markers [4,5]. Microsatellite analyses of both sire and dam lines indicate that the Polish Konik has high genetic variability and no significant inbreeding. For example, the research [4,5] report large allelic richness (~6.5–7.6 alleles per locus) and high heterozygosity (He ≈ 0.70). Mitochondrial DNA studies [6,8] similarly reveal many maternal haplotypes (43 and 19 observed, accordingly) across multiple haplogroups (A, B, E, G, J, R, etc.), with overall haplotype diversity ≈ 0.96 and ≈0.92, accordingly.

While mitochondrial DNA studies give information about maternal lineages, they provide only limited insight into the genome. Microsatellite markers provide a view of genetic variation both within and among populations but without the resolution for evolutionary analysis.

The present study integrates modern genomic techniques with current knowledge about the breed’s evolutionary analysis to provide new insights into the origin and phylogeny of the Polish Konik horse. Furthermore, the obtained whole-genome sequencing (WGS) data provide a basis for clarifying the relationship between the Polish Konik and the extinct Tarpan, and for shaping future conservation strategies. Recent genomic research has challenged the long-held view of the Tarpan as the direct wild ancestor of modern horses. Librado et al. [9] demonstrated that the individual labeled as Tarpan was admixed, with ~33% ancestry from a native European (Corded-Ware-associated) lineage and ~67% from the steppe DOM2 lineage (DOM2 refers to the main domestic horse lineage that spread from the Eurasian Steppe, into which modern breeds fall), which ultimately contributed to the ancestry of modern domestic horses. Thus, the Tarpan should not be regarded as a pure DOM2 clade. We describe it as having mixed origins, with its indigenous European component progressively diluted by incoming DOM2 horses from the Pontic–Caspian steppe (~4200 ybp). Moreover, Librado et al. [10] reported a second low-coverage Tarpan genome that likewise retains a high proportion (~45%) of Corded-Ware-related ancestry. These results have refuted the traditional hypothesis of the Tarpan as a direct progenitor of modern horses or as a hybrid with the Przewalski horse.

In light of recent findings redefining the evolutionary position of the Tarpan, we analyzed a broad panel of samples to explore its genetic connection with the Polish Konik, a breed historically believed to descend directly from it. The dataset included 12 Polish Koniks (six mares and six stallions), and a reference genome from an equine individual referred to as Tarpan. To provide robust comparative context, we additionally used 148 whole-genome sequences publicly available in the European Nucleotide Archive (ENA) and in the DRUM database, representing a wide range of modern horse breeds. This comprehensive dataset allowed for an in-depth genomic comparison across breeds, enabling a re-evaluation of the genetic affinities between the Polish Konik, the extinct Tarpan, and other equine breeds.

## 2. Materials and Methods

### 2.1. Sampling

#### 2.1.1. Polish Konik

In the present study, 12 samples of the Polish Konik belonging to different dam and sire lines were examined (Table 1). The individuals were selected using studbook records with the explicit aim of minimizing close relatedness. Selection prioritized animals from different studs, distinct dam and sire lines and avoided pairs sharing common ancestors within at least four generations. The lines were assigned to all the horses based on pedigree data available online at www.baza.pzhk.pl accessed on 28 April 2026. From all 16 dam lines present in the studbook, we selected individuals belonging to 8 lines with the largest number of individuals (4) belonging to the Liliputka I line—known as the oldest Polish Konik dam line [11] (www.baza.pzhk.pl accessed on 28 April 2026). Among the sire lines, there were specimens belonging to 5 out of 6 Polish Konik sire lines. As the material for this study, we used blood samples that were collected during routine parentage verification analysis and stored at a genetic material bank of the National Research Institute of Animal Production in Poland.

#### 2.1.2. Previously Published Ancient and Modern Horse Genomes

To collect comparative genomic data, we used 148 whole genome sequences previously published in the European Nucleotide Archive database and in the DRUM database (University of Minnesota) by Durward-Akhurst et al. [12]. The total list of samples is available in the Supplement (Data S1). The analysis included modern horses belonging to different horse breeds representing different origins: Thoroughbred (TH: 13 individuals), Arabian (AR: 11), Franches-Montagnes (Franches: 11), Standardbred (SB: 10, USA), Warmblood (WB: 10), Belgian (BG: 10), Morgan (Morg: 10, USA), French Trotter (FT: 10), Icelandic (9), Clydesdale (Clyd: 9), Jeju (JP: 9), Westphalian (WP: 9), Halfinger (8), Connemara (CP: 8), Dülmener (SRR1048526: 1), Exmoor (ERR11180374: 1), and Sorraia (SRR2142313: 1), as well as two Przewalski horses (SRR1564424, ERR982714), the Tarpan sequence (ERR6466048) and five ancient DNA sequences from Poland (ERR6798756, ERR6798762, ERR6465807, ERR6465812, ERR6465818). The Tarpan genome, originally published by Librado et al. [9], represents a late historic individual identified as a Tarpan and sequenced using ancient DNA protocols. The average sequencing coverage of this genome is low (below 1×), which limits confident genotype calling, particularly at heterozygous sites. As a result, genotype calls derived from this genome were interpreted cautiously in downstream analyses. The Polish ancient DNA sequences come from the bones collected in Kazimierza Wielka and Miechów settlements, which are dated to the Early Bronze Age (1900–1200 BCE) and Eneolithic Funnel Beaker Culture (~2500 years ago), creating a genetic picture of the horse living in Poland in ancient times.

### 2.2. WGS of Polish Konik Horses

The DNA extraction was performed using the Sherlock AX kit (A&A Biotechnology, Gdańsk, Poland) with 100 μL of whole blood. The samples were prepared for extraction by incubation at 50 °C with the lysis buffer and proteinase K. Next, samples were processed using filtration and spin columns, and the eluted DNA was precipitated with isopropanol. Pellets were washed with 70% ethanol, dried, and dissolved in 30 μL of TE buffer. DNA quality was determined by checking concentration and purity on the NanoDrop 2000 spectrophotometer (Thermo Fisher Scientific, Waltham, MA, USA).

Libraries for WGS were constructed for all 12 individuals. Following indexing, the samples were grouped into four pooled sets to facilitate multiplex sequencing (K1–K3—1 pool; K4–K6—2 pool; K7–K9—3 pool; and K10–K12—4 pool). Each prepared pool was subject to quality control, which included three main steps: concentration measurement (NanoDrop), size measurement (Bioanalyzer 2100, Fragment Analyzer, Agilent Technologies, Santa Clara, CA, USA), and mimicking cluster creation (RT-PCR). All the libraries met the quality standards and were sequenced on the NovaSeq S4 Analyser (Illumina, San Diego, CA, USA).

### 2.3. Bioinformatic Analysis

#### 2.3.1. Preprocessing and Variant Calling

The WGS data of the four pools representing Polish Konik horses, together with the publicly available reference panel, were processed using a standardized pipeline based on the Genome Analysis Toolkit (GATK v4.2.0) [13]. To integrate ancient genomes, we applied conservative filters and avoided genotype-level overinterpretation for low-coverage samples. Raw paired-end reads underwent quality control using FastQC v0.11.9 [14] to assess base-call quality, read length distribution and adapter contamination. The average read length of the newly generated Polish Konik data was (paired-end 2 × 150 bp on the Illumina NovaSeq S4). Low-quality bases and adapter sequences were trimmed using Trimmomatic v0.39 [15] with parameters ILLUMINACLIP:TruSeq3-PE.fa:2:30:10 LEADING:3 TRAILING:3 SLIDINGWINDOW:4:20 MINLEN:50. High-quality reads were aligned to the EquCab3.0 reference genome (GenBank assembly accession GCF_002863925.1) using BWA-MEM v0.7.17 [16] with default parameters. Alignments were sorted and indexed with SAMtools v1.15. PCR and optical duplicates were marked with Picard MarkDuplicates v2.27 [17]. Base quality score recalibration (BQSR) was performed in two passes using GATK BaseRecalibrator and ApplyBQSR, using [known-sites VCF reference version 111 from Ensembl] as the known-variants resource. Average per-pool depth of coverage after deduplication was 33.6× for the newly sequenced Polish Konik samples and 32.3× for the reference panel samples (with the exception of ancient samples which had low coverage—from 1 to 4× for ancient samples, 15× for Tarpan and 78× for Exmoor).

Variant calling was performed using GATK’s HaplotypeCaller (-ERC GVCF, ploidy = 2 for individual samples; for the four pooled Polish Konik libraries the -ploidy [N] option was set to reflect the number of individuals per pool) to produce per-sample gVCF files. Joint genotyping was conducted with GenotypeGVCFs followed by GenotypeGVCFs (default parameters). The resulting multi-sample VCF was filtered using VariantFiltration applying GATK best-practices hard filters for SNPs (QD < 2.0 || FS > 60.0 || MQ < 40.0 || MQRankSum < −12.5 || ReadPosRankSum < −8.0 || SOR > 3.0) and additional thresholds of QUAL > 30 and DP > 10, after which only biallelic SNPs marked PASS were retained for downstream analyses.

#### 2.3.2. Data Transformation and Quality Filtering

The filtered VCF files were converted to PLINK [18] format (PED/MAP) using the GATK VariantsToTable tool and PLINK v1.9. Quality control in PLINK involved filtering variants with a minor allele frequency (MAF) below 0.05, genotyping rate lower than 80%, and Hardy–Weinberg equilibrium (HWE) *p*-value less than 1 × 10^−6^. Additionally, samples with more than 20% missing genotypes were excluded. These thresholds were chosen as a compromise between retaining enough informative markers for population-level analyses and excluding poorly supported loci. Because the dataset combines modern genomes with ancient low-coverage and pooled samples, a stricter missingness threshold would have substantially reduced the number of shared markers available for downstream analyses. The data obtained for the Polish Konik breed was then merged with the previously calculated data for other breeds, and then the common markers were used for further analysis.

#### 2.3.3. Admixture Analysis

Population structure was further investigated using ADMIXTURE v1.3.1 [19]. The analysis was conducted for K values ranging from 1 to 16 to infer the most probable number of ancestral populations. The optimal K was determined by cross-validation (CV) error, which was calculated for each run. The admixture proportions were visualized in a bar plot to depict the ancestral components across the Polish Konik and other horse breeds.

#### 2.3.4. Principal Component Analysis (PCA)

To explore population structure, a PCA was performed using the filtered SNP data in PLINK. The eigenvalues and eigenvectors were computed using the --pca option to identify the major axes of genetic variation. The first two principal components (PC1 and PC2) were visualized to assess clustering among Polish Konik and other breeds. As an additional check, we also performed PCA using one representative individual per breed to balance the sample sizes across groups (Figure S1). This down-sampled PCA yielded a similar pattern of breed clustering, indicating our main results are robust to uneven sample counts.

#### 2.3.5. Neighbor-Joining Tree and Bootstrap Analysis

Genetic relationships among individuals were visualized using a neighbor-joining (NJ) tree constructed from genome-wide SNP data. Prior to tree construction, SNPs were filtered and pruned for linkage disequilibrium using PLINK v1.9 with the parameters indep-pairwise 50 5 0.2 to reduce the impact of linked markers on distance estimation. The resulting pruned SNP dataset was used to compute pairwise genetic distances among individuals. The NJ tree was reconstructed using the distance-based neighbor-joining algorithm implemented in the Biopython phylogenetics toolkit. To assess the robustness of the inferred topology, bootstrap analysis was performed by resampling SNP loci with replacement from the LD-pruned genotype matrix. For each bootstrap replicate, a new distance matrix was calculated, and an NJ tree was reconstructed. Bootstrap support values were calculated from 1000 replicate datasets and mapped onto the reference NJ topology. Because genome-wide SNP datasets from closely related populations primarily reflect patterns of genetic similarity rather than strict phylogenetic relationships, the NJ tree should be interpreted as a representation of population structure rather than a fully resolved evolutionary phylogeny.

#### 2.3.6. Visualization of the Results

All plots and graphs were generated using R (v4.2.0) and ggplot2. PCA plots, NJ trees, and admixture bar plots were prepared to illustrate genetic relationships and population structure.

## 3. Results

Variant calling from the 12 Polish Konik samples (KP, in four pools) resulted in the identification of 8,830,066 SNPs and 475,842 INDELs after applying the filtration procedure. In contrast, the analysis of all other breeds, comprising 148 samples, yielded a total of 31,249,737 variants post-filtration, including 30,624,742 SNPs and 624,995 INDELs. Simple summary statistics for KP are presented in Table S1. The variant data from both groups were subsequently merged into a single file, filtered to retain common markers, and then utilized for all downstream analyses.

**Figure 1:**
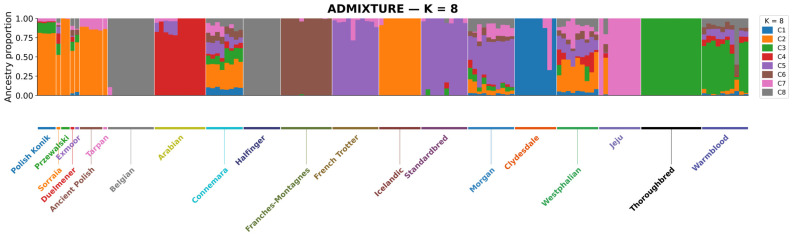
The visualization of eight clusters representing different ancestral components. Different colors represent composition of ancestral components in four Polish Konik and 148 horse genomes from the other breeds.

To identify genetic clusters and estimate the proportion of an individual’s genome derived from each cluster, admixture analysis (ADMIXTURE v1.3.1.) was performed. A set of simulations of the number of clusters (K) was made for every K between 1 and 16. The CV error reached the lowest value with K = 8 (Figure S2), indicating that the genetic variation in the entire dataset is best represented by eight ancestral components (the comparison of ADMIXTURE results for K = 2, K = 4, K = 6 and K = 8 is showed on Figure S3). Therefore, this number of ancestral clusters was chosen to describe the genetic structure of the studied populations, and the visualization of the clusters is shown in Figure 1.

**Figure 2:**
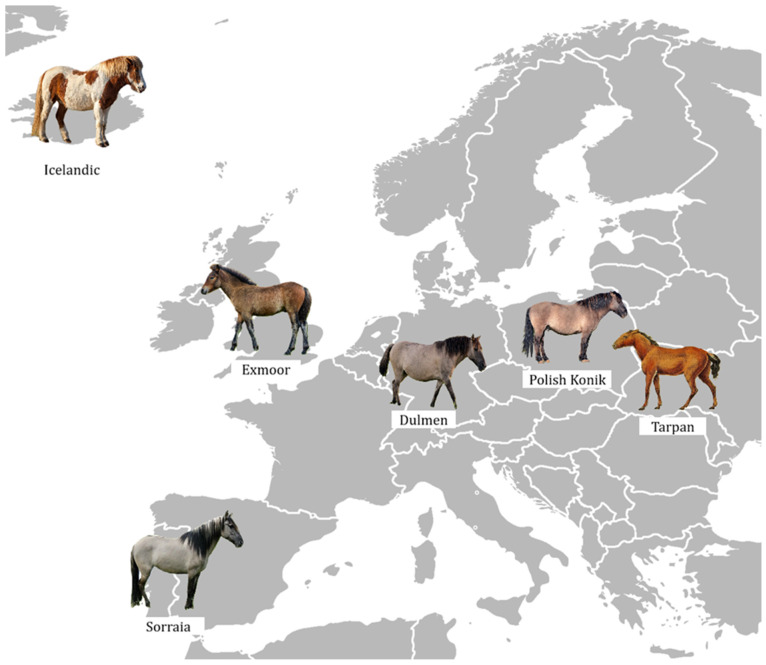
The occurrence of horse breeds that share the predominant Cluster 2 with Polish Konik.

Admixture analysis of the population structure revealed that the Polish Konik breed is predominantly composed of Cluster 2 (orange ~77%), with minor contributions from Cluster 3 (green ~16%) and Cluster 7 (pink ~5%). This pattern suggests a largely similar ancestry, strongly dominated by Cluster 2, with only limited introgression from other genetic components. The genetic signature also includes all ancient horse samples from Poland, as well as Tarpan and several primitive pony breeds such as Dülmener, Sorraia, Exmoor, and Icelandic (Figure 2). These populations share ancestry components with ancient and primitive populations.

Among the investigated populations, breeds showing moderate to low representation of Cluster 2, and thus partial shared components with the Polish Konik, include Franches-Montagnes, Standardbred, and Warmbloods, each exhibiting a certain degree of admixture (exact proportions vary by individual). Breeds showing only limited admixture with Polish Konik include Thoroughbred (TB), Arabian (AR), and Clydesdale (CL). The final group of breeds shows no significant shared ancestry components with the Polish Konik based on the admixture analysis. These include Belgian, Connemara, French Trotter, and Haflinger, which were each dominated by distinct clusters without any appreciable Cluster 2 component.

**Figure 3:**
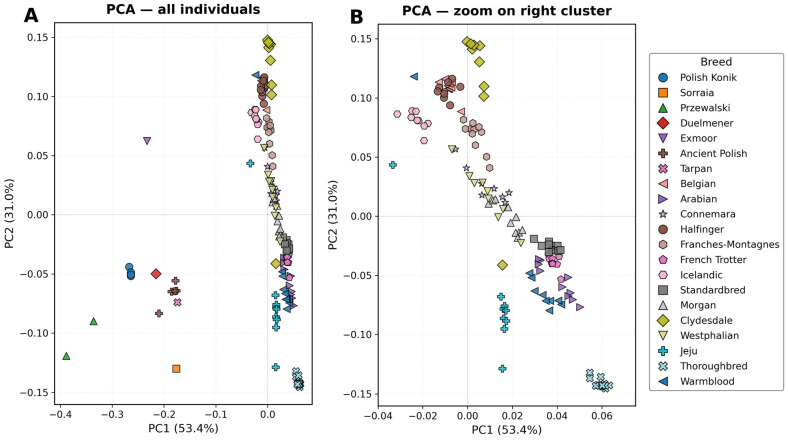
The PCA plot of all analyzed horses. Two views on the PCA plot show: the plot of all horses included in the research (plot (**A**)), and a magnified section of the graph (panel (**B**)) illustrating minor differences between individuals. The PC1/PC2 axes are labeled with the percentage of variance explained. The PC1 and PC2 components present the position of Polish Konik horses in relation to the other analyzed horses. PC1 and PC2 are the first two principal components capturing the largest and second-largest genetic variance, respectively. These axes summarize genetic differences but do not correspond to specific biological traits.

The PCA plot of different horse populations (Figure 3, Data S2) shows the grouping of the analyzed breeds on the plane defined by the principal components (PC1 and PC2). The PCA plot supports findings from the admixture analysis and shows a clear separation among sampled groups along major axes of genetic variation (PC1 and PC2). On the PCA graph, Polish Konik individuals (blue dots) form a cluster located in the central-left region, indicating a high degree of genetic homogeneity (relatively low within-sample diversity). This clustering pattern aligns with the admixture results, where the breed is predominantly composed of Cluster 2 (orange), with minor contributions from Cluster 3 (green) and Cluster 7 (pink). Such composition reflects a largely uniform one, with only limited introgression from other clusters. Directly adjacent to the right of the Polish Konik cluster, we observed a grouping of individuals representing ancient horse samples, Dülmener, Sorraia, and Icelandic ponies. These breeds also displayed strong proportions of Cluster 2 in the admixture plot, sharing genetic similarity with the Polish Konik. This group appeared to reflect a preserved ancestral genetic component common among several primitive and Northern Eurasian horse lineages. In the lower-left corner of the PCA plot are Przewalski horses, characterized by significant genetic distinctiveness from most modern breeds. It is also worth noting the grouping of the Tarpan sequence with the sequences of ancient horses from Poland.

The Polish Konik horses, along with other primitive breeds (Dülmener pony, Sorraia pony), ancient horses of Poland, and Tarpan, are grouped in the lower-left quadrant, with negative PC1 and PC2 values, and are located close to each other in the PCA space. The Exmoor pony is also located not far from them but at a noticeable distance. The observed proximity of Polish Konik individuals to some ancient samples and to Przewalski horses in the PCA space indicates shared genetic components but does not by itself demonstrate preservation of pre-domestication traits. Such clustering can reflect various processes, such as shared ancestry, but also retention of DOM2-derived diversity, or sampling effects.

To verify that the clustering observed in the full PCA was not driven by the uneven sample counts across breeds, we performed an additional PCA on a down-sampled dataset in which one individual per breed was randomly selected (n = 21 in total, using a fixed random seed for reproducibility; Supplementary Figure S1). The down-sampled PCA recovered the same qualitative structure as the full analysis: Polish Konik individuals remained located in the lower-left region of the plot alongside ancient Polish, Tarpan, Dülmener, Sorraia and Exmoor, and were clearly separated from the modern sport and draft breeds along PC1 and PC2. Przewalski horses retained their characteristic position in the lower-left corner. This indicates that the position of Polish Konik relative to the ancient and primitive horse cluster does not depend on the larger sample sizes of the modern reference breeds and is therefore robust to sample-size imbalance.

The neighbor-joining tree reconstructed from genome-wide SNP data broadly reflected the genetic relationships among the analyzed horse populations. Individuals belonging to the same breed generally clustered together, indicating clear genetic differentiation between most breeds. For example, individuals from the same population tended to form compact clusters, consistent with their shared genetic background. Bootstrap support values varied across the tree. As expected for distance-based trees derived from genome-wide SNP data among closely related domestic populations, many deeper nodes showed relatively low bootstrap support. This reflects the limited phylogenetic signal separating recently diverged breeds and the influence of historical gene flow among domestic horse populations. Consequently, the NJ tree should be interpreted primarily as a visualization of genetic similarity and population structure rather than as a strict representation of evolutionary branching order.

Nevertheless, several breed-specific clusters were consistently recovered across bootstrap replicates, supporting the genetic distinctiveness of these populations within the dataset.

**Figure 4:**
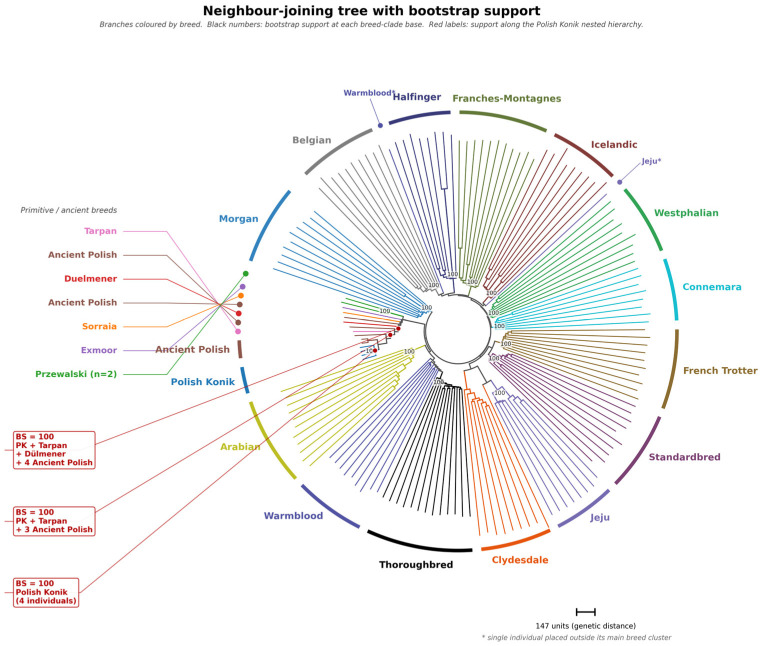
The circular neighbor-joining phylogenetic tree of the 152 analyzed horses with bootstrap analysis performed (1000 replicates).

**Figure 5:**
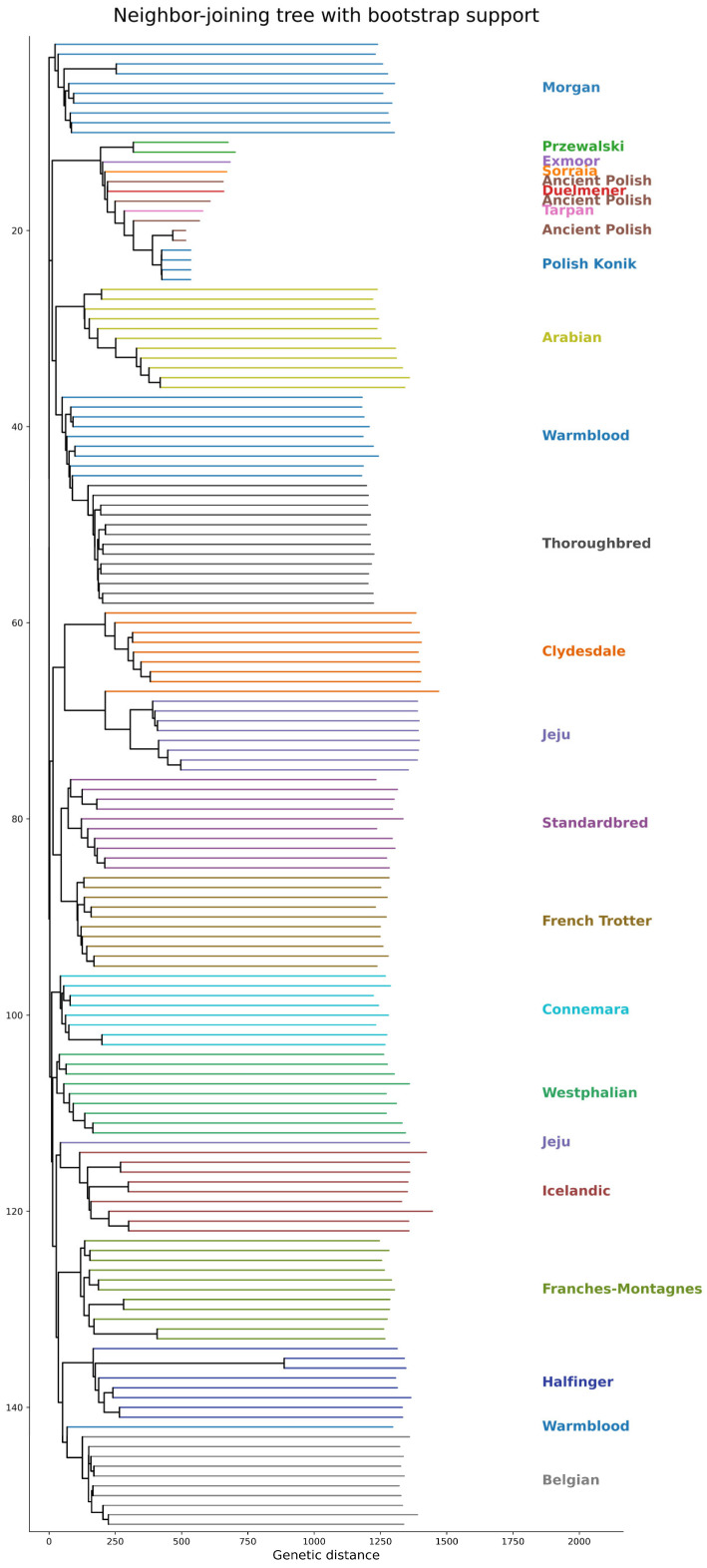
The rectangular neighbor-joining phylogenetic tree of the 152 analyzed horses with bootstrap analysis performed (1000 replicates).

The phylogenetic trees (Figure 4 and Figure 5) were constructed based on 152 horse samples, including four Polish Konik pools, Tarpan sequence, and ancient equine genomes. An additional figure (Figure S4) representing each individual on the phylogenetic trees is available in the supplement. The NJ tree estimates the minimum evolutionary distance required to explain the genetic differences among the analyzed horses. Polish Konik individuals form a distinct and tight clade, grouped closely with several Welsh Pony (WP) and Warmblood (WB) individuals, as well as ancient samples such as Sorraia, Przewalski, and Dülmener pony. This pattern suggests genetic similarity among these groups. The clustering of sequences into separate branches effectively reflects their genetic relationships. The placement of Polish Konik horses alongside other specific breeds implies a closer genetic relationship between them. Notably, primitive breeds and ancient samples, including Dülmener, Sorraia, and Icelandic horses, form nearby but separate clades, indicating a close genetic similarity with the Polish Konik. In particular, Polish Koniks are clustered together with Dülmener ponies, Jeju ponies, Morgan horses, and Westphalian horses, further supporting their link to both primitive and historically conserved equine lineages. In contrast, Polish Konik horses are separated from wild and ancient horse lineages, such as the Tarpan, and several ancient Polish horses, which occupy distant branches of the tree. Similarly, modern horse breeds—including Thoroughbred (TH), Connemara Pony (CP), and Warmblood (WB)—form a separate and distinct clade, highlighting their genetic divergence with the Polish Konik. These modern breeds are also genetically distinct from coldblood breeds, such as the Clydesdale and Belgian horses.

## 4. Discussion

In our study, WGS data enabled genetic analyses that provided new insights into the ancestry and genetic structure of the Polish Konik, shedding new light on its evolutionary origins, its relationship to the extinct Tarpan, and its position among modern horse breeds. The genetic composition of the Polish Konik suggests a largely similar ancestry, dominated by a single ancestral component, with only limited introgression from other genetic backgrounds. Notably, this genetic signature is also shared with ancient horse samples from Poland, as well as with primitive pony breeds such as the Dülmener, Sorraia, Exmoor, and Icelandic [20]. These findings are further supported by the PCA plot, where Polish Konik individuals form a compact and distinct cluster, reflecting their relatively low within-sample diversity. This placement closely aligns with breeds showing similar admixture patterns, particularly primitive and ancient horses, which cluster immediately adjacent to the Polish Konik breed. This finding indicates that Polish Konik genomes share ancestry components with several ancient and relatively unadmixed European lineages. However, genetic proximity in PCA or shared ADMIXTURE components does not necessarily imply retention of primitive or pre-domestication phenotypes; such shared components may instead reflect broad DOM2-related diversity or other historical admixture events. These patterns are consistent with deeper historical processes, including multiple migrations and population replacements that reshaped horse populations across Europe [21].

The additional genetic components observed in the Polish Konik were associated with Warmblood, Westphalian, and Thoroughbred populations, indicating a degree of admixture (ancestral contributions detectable when comparing across breeds and ancient samples) with modern horse breeds. This admixture likely reflects historical breeding practices and interactions that have contributed to the breed’s current genomic diversity and phenotypic variation [22]. Importantly, these findings are consistent with previous studies highlighting the influence of selective breeding on the genetic makeup of the Polish Konik [23].

The NJ tree indicates that the Polish Konik shares certain genetic similarities with modern warmblood and pony breeds. Notably, both the NJ tree and the PCA plot consistently reveal a close genetic relationship between the Polish Konik and the Dülmener pony, suggesting shared ancestry (reflecting genetic similarity), though PCA and NJ alone do not prove historical gene flow. It is worth highlighting that the Dülmener pony appears the closest relatively to the Polish Konik on the PCA plot. This observation is consistent with the analysis of Y chromosome markers [6]. This affinity is further supported by all conducted genetic analyses and is not coincidental. Rather, it reflects a well-documented historical connection. In the 1950s, Polish Konik stallions were purposefully introduced into the Dülmener breeding program in Germany to reintroduce primitive traits and eliminate domesticated characteristics from the population. One of the most notable examples is the stallion Nugat XII, who was used to breed Dülmener mares between 1957 and 1963 [24]. This targeted crossbreeding effort has left a lasting genetic imprint, which remains evident in the modern Dülmener pony and accounts for its close genetic relationship with the Polish Konik.

The Polish Konik is considered a unique and valuable breed, possibly descended from wild horses that once lived in Europe. If this origin is confirmed, it would make the breed a rare example of a preserved ancestral horse type. While the origin of this hypothesis is well-documented, the historical narrative surrounding the breed’s ancestry has often been accompanied by subjective interpretations and speculative commentary from various authors seeking an alternative lineage of origin for the Polish Konik [2,25]. Historical records indicate that Samuel Georg Gmelin described free-roaming horses in the Bobrovsk region of Russia in the 18th century and identified them as “wild”. The term “Tarpan” was later established in the mid-19th century.

In this study, WGS was employed to generate high-resolution genetic data used in various analyses aimed at uncovering new insights into the origins and genetic structure of the Polish Konik horses. Modern molecular approaches, such as WGS, offer significant potential for exploring genetic relationships among horse breeds and provide powerful tools to investigate breed histories and evolutionary backgrounds. Unlike earlier genetic methods that focused on specific genome regions, such as mitochondrial DNA or selected microsatellite markers, WGS enables comprehensive, full-genome coverage, allowing for a more detailed exploration of population structure, ancestral relationships, and genomic diversity. This broader perspective is especially valuable for breeds like the Polish Konik, where clarifying the ancestry and genetic distinctiveness is critical for both scientific understanding and practical conservation [26]. Knowledge about the genetic profile of the Polish Konik is particularly important in light of its growing role in ecological restoration and rewilding programs [27]. Maintaining genetic purity and primitive traits remains key to conservation, with WGS results offering clarity on breed origins and guiding informed preservation efforts. The groundbreaking research of Librado et al. [9], using shotgun sequencing of 264 ancient horse genomes, fundamentally reshaped our understanding of horse domestication. Their findings revealed that all modern domestic horses descend from a single ancestral population known as DOM2, which emerged in the Western Eurasian steppes, specifically the lower Volga–Don region, around the late fourth to early third millennium BCE. This DOM2 lineage rapidly replaced nearly all pre-existing local populations across Eurasia by ~2000 BCE, a process driven by the widespread use of horses in transport and warfare. Crucially, this study refuted earlier assumptions that Tarpans represented a wild ancestral form of modern horses. Instead, Tarpans were shown to be admixed individuals, resulting from gene flow between European native horses (including those linked to the Corded Ware Culture) and DOM2-related horses, with an estimated 28.8–34.2% CWC ancestry. This challenges long-held narratives that depicted Tarpans either as a direct wild progenitor of domestic breeds or as feral DOM2 descendants. In the context of our study, these results are pivotal in that they provide critical genomic evidence that helps to clarify the origins of primitive breeds such as the Polish Konik, offering a framework to reassess earlier historical and morphological hypotheses with high-resolution molecular data.

Our results do not support the hypothesis that the Polish Konik is a direct descendant of the Tarpan. Although early studies of body morphology suggested a close relationship between the two [28], modern genomic techniques offer a more detailed view of the breed’s origins. This study reveals that the Polish Konik has retained genetic traits associated with ancient and primitive horse populations, but also shows admixture with modern horse breeds, underlining the complexity of its origins. This viewpoint is consistent with recent studies that question the direct lineage between the Polish Konik and the Tarpan, highlighting how selective breeding and historical narratives have influenced our understanding of the breed’s origins [25].

The applications of our findings are not limited to historical and evolutionary aspects because the understanding of the Polish Konik genetics is crucial also for conservation efforts, especially maintaining genetic diversity and its primitive traits. Future studies, especially using the WGS method, can increase our knowledge of the breed, and lead to more effective conservation strategies. The connection of modern genomic techniques with historical and morphological studies will be the most effective way to present clearer information about the Polish Konik’s origins, which will benefit both scientific research and the management of this unique breed of horses.

A complementary direction for future work would be to compare patterns of within-breed genetic diversity—for example the number of segregating SNPs, observed heterozygosity, or runs of homozygosity—across the breeds analyzed here. Such comparisons are biologically informative as they reflect long-term effective population size, demographic history, and the intensity of artificial selection applied to each breed. In the present study, however, the comparative panel is strongly imbalanced in sample size, ranging from a single individual for Sorraia, Dülmener, Exmoor and Tarpan to thirteen for Thoroughbred, and the ancient samples were sequenced at low coverage (1–4×). Because the number of within-breed segregating SNPs scales strongly with both sample size and sequencing depth, direct per-breed SNP comparisons on a dataset of this composition would primarily reflect technical differences rather than true biological diversity, and we therefore restricted the present analysis to the common-dataset comparison. Future studies based on balanced, deeply sequenced panels—including a larger number of Polish Konik individuals from all dam and sire lines, as well as multiple representatives of the primitive pony breeds—will allow these breed-level diversity metrics to be estimated and compared in a statistically robust way, and will further refine the picture of how Polish Konik genetic diversity sits within the broader landscape of European primitive horse breeds.

### Limitations of the Study

This analysis has several limitations that should be considered when interpreting the results. Firstly, the Polish Konik sample size was relatively small. Although individuals were chosen to minimize recent relatedness, the limited sample size reduces statistical power and restricts the generalizability of breed-wide inferences. Furthermore, breeds in the comparative panel are represented by uneven sample counts, which can bias clustering and ancestry estimates. To assess the impact of this imbalance on the PCA results, we performed an additional balanced PCA based on one individual per breed (Supplementary Figure S1); the resulting clustering pattern was qualitatively identical to that of the full analysis, with Polish Konik remaining grouped with the ancient and primitive horse samples and clearly separated from modern sport and draft breeds. This indicates that the main PCA structure reported here is not driven by sample-size imbalance, although we caution that ADMIXTURE estimates and fine-scale breed-to-breed comparisons should still be interpreted with the uneven sample composition in mind. Secondly, applying programs like ADMIXTURE, which are designed to work on the individual level, may introduce biases in pooled or mixed data. For our case, the pooled samples were analyzed alongside individual-level data from other breeds. Pooling can mask individual variation which may affect the estimates of ancestry and population structure accuracy. An additional limitation concerns the inclusion of low-coverage ancient genomes, including the Tarpan individual. At sequencing depths below ~1×, heterozygous positions are frequently underrepresented, and many rare or lineage-specific variants may remain undetected. Consequently, the ability to detect Tarpan-specific genetic signals may be reduced, potentially attenuating fine-scale ancestry inferences involving ancient samples. For the same reason, we did not perform direct per-breed comparisons of segregating SNP counts; such comparisons would be confounded by sample size and by the low coverage of the ancient samples (1–4×), and would primarily reflect technical differences rather than biological diversity.

## 5. Conclusions

The presented genetic analysis of Polish Konik horses provides new information on the origins and genetic structure of the breed. The WGS method and the applied genetic analysis, including admixture analysis, PCA plot, and NJ tree, showed diverse and partially overlapping results. Our analyses suggest that the Polish Konik shares genomic components with several ancient and primitive-type horse samples, while also showing signals consistent with admixture from modern breeds. These patterns are indicative of a complex origin of the Polish Konik. The observed close affinity to the Dülmener pony and the absence of a unique, stronger relationship to the published Tarpan genome in our dataset are consistent with a multifaceted breed history. Our results do not support the hypothesis that the Polish Konik is a direct descendant of the Tarpan; however, due to the low coverage of the Tarpan genome and the pooling of Polish Konik samples, this conclusion should be interpreted with caution.

## Figures and Tables

**Table 1:** Information about selected individuals of Polish Konik horses. The information includes dam and sire lines of the selected horses (in parentheses the year of the line creation, understood as the oldest individual appearing in written sources, in the Polish Konik studbook), and the no. of DNA pools used in analysis).

Sample	Pool	Sex	Birth	Dam Line	No. of Dam Generation	Sire Line	No. of Sire Generation
K1	1	♀	2001	Liliputka I (1928)	10	Wicek (<1930)	9
K2		♀	1999	Liliputka I (1928)	8	Liliput (1920)	7
K3		♀	2005	Liliputka I (1928)	12	Goraj (1935)	9
K4	2	♀	1991	Tarpanka I (1937)	7	Wicek (<1930)	7
K5		♀	1991	Zaza (1933)	8	Chochlik (1940)	5
K6		♀	2010	Wola (1943)	10	Liliput (1920)	9
O1	3	♂	2007	Liliputka I (1928)	11	Wicek (<1930)	8
O2		♂	2008	Traszka (-)	7	Wicek (<1930)	9
O3		♂	2009	Traszka (-)	9	Wicek (<1930)	9
O4	4	♂	2008	Urszulka (1934)	9	Chochlik (1940)	6
O5		♂	2009	Ponętna (1946)	10	Goraj (1935)	10
O6		♂	2008	Karolka (1933)	10	Glejt I (1944)	9

## Data Availability

The obtained WGS sequences of Polish Konik horses were deposited in the Sequence Read Archive (SRA) database and received SubmissionID: SUB15643985 and BioProject ID: PRJNA1331525. The other data is provided within the manuscript, Supplementary Information files and upon request from the author.

## Supplementary Materials

The following supporting information can be downloaded at: https://www.mdpi.com/article/10.3390/ani16111669/s1, Figure S1: The PCA plot performed on a down-sampled dataset in which one individual per breed was randomly selected (n = 21 in total); Figure S2: CV error across different values of K. The figure illustrates the CV error associated with varying values of K in the k-nearest neighbors (KNN) algorithm. The error is reaching a minimum at K = 8; Figure S3: The ADMIXTURE results for K = 2, K = 4, K = 6 and K = 8; Figure S4: The neighbor-joining phylogenetic tree of the 152 analyzed horses with bootstrap analysis performed (1000 replicates) and with all the samples visualized; Table S1: Summary statistics for sequencing of the pools; Data S1: List of analyzed samples; Data S2: Principal Component Analysis values of analyzed horses. The figure displays two-dimensional principal component analysis (PCA) values based on the first two principal components (PC1 and PC2). The distribution of points along PC1 and PC2 highlights clustering patterns that correspond to genetic or population structure, with certain groups forming distinct clusters, while others exhibit partial overlap.

## Abbreviations

The following abbreviations are used in this manuscript:

NJ	Neighbor-Joining
PCA	Principal Component Analysis
WGS	Whole-Genome Sequencing

## References

[1] Pasicka E. (2013). Polish Konik horse—Characteristics and historical background of native descendants of Tarpan. Acta Sci. Pol. Med. Vet..

[2] Van Vuure C.T. (2014). On the origin of the Polish Konik and its relations to Dutch nature management. Lutra.

[3] Vetulani T. (1948). O regeneracji tarpana leśnego w Puszczy Białowieskiej. Pamiętnik XXI Zjazdu Państwowej Rady Ochrony Przyrody.

[4] Fornal A., Kowalska K., Ząbek T., Piestrzynska-Kajtoch A., Musiał A.D., Ropka-Molik K. (2020). Genetic diversity and population structure of Polish Konik horse based on individuals from all the male founder lines and microsatellite markers. Animals.

[5] Fornal A., Kowalska K., Ząbek T., Piestrzynska-Kajtoch A., Musiał A.D., Ropka-Molik K. (2021). Genetic variability and population structure of Polish Konik horse maternal lines based on microsatellite markers. Genes.

[6] Musiał A.D., Radović L., Stefaniuk-Szmukier M., Bieniek A., Wallner B., Ropka-Molik K. (2024). Mitochondrial DNA and Y chromosome reveal the genetic structure of the native Polish Konik horse population. PeerJ.

[7] Kamiński S., Hering D.M., Jaworski Z., Zabolewicz T., Rusc A. (2017). Assessment of genomic inbreeding in Polish Konik horses. Pol. J. Vet. Sci..

[8] Cieślak J., Wodas Ł., Borowska A., Cothran E.G., Khanshour A.M., Mackowski M. (2017). Characterization of the Polish Primitive Horse (Konik) maternal lines using mitochondrial D-loop sequence variation. PeerJ.

[9] Librado P., Khan N., Fages A., Kusliy M.A., Suchan T., Tonasso-Calvière L., Schiavinato S., Alioglu D., Fromentier A., Perdereau A. (2021). The origins and spread of domestic horses from the Western Eurasian steppes. Nature.

[10] Librado P., Tressières G., Chauvey L., Fages A., Khan N., Schiavinato S., Calvière-Tonasso L., Kusliy M.A., Gaunitz C., Liu X. (2024). Widespread horse-based mobility arose around 2200 BCE in Eurasia. Nature.

[11] Szwaczkowski T., Greguła-Kania M., Stachurska A., Borowska A., Jaworski Z., Gruszecki T.M. (2016). Inter- and intra-genetic diversity in the Polish Konik horse: Implications for the conservation program. Can. J. Anim. Sci..

[12] Durward-Akhurst S.A., Marlowe J.L., Schaefer R.J., Springer K., Grantham B., Carey W.K., Bellone R.R., Mickelson J.R., McCue M.E. (2024). Predicted genetic burden and frequency of phenotype-associated variants in the horse. Sci. Rep..

[13] Van der Auwera G.A., O’Connor B.D. (2020). Genomics in the Cloud: Using Docker, GATK, and WDL in Terra.

[14] Andrews S. (2010). FastQC: A Quality Control Tool for High Throughput Sequence Data.

[15] Bolger A.M., Lohse M., Usadel B. (2014). Trimmomatic: A flexible trimmer for Illumina sequence data. Bioinformatics.

[16] Li H. (2013). Aligning sequence reads, clone sequences and assembly contigs with BWA-MEM. arXiv.

[17] Broad Institute (2019). Picard Toolkit.

[18] Purcell S., Neale B., Todd-Brown K., Thomas L., Ferreira M.A.R., Bender D., Maller J., Sklar P., De Bakker P.I., Daly M.J. (2007). PLINK: A tool set for whole-genome association and population-based linkage analyses. Am. J. Hum. Genet..

[19] Alexander D.H., Novembre J., Lange K. (2009). Fast model-based estimation of ancestry in unrelated individuals. Genome Res..

[20] Duderstadt S., Distl O. (2024). Genetic diversity and population structure of Dülmen Wild, Liebenthal and Polish Konik horses in comparison with Przewalski, Sorraia, German Draught and Riding Horses. Animals.

[21] Bendrey R. (2012). From wild horses to domestic horses: A European perspective. World Archaeol..

[22] Bozlak E., Radovic L., Remer V., Rigler D., Allen L., Brem G., Stalder G., Castaneda C., Cothran G., Raudsepp T. (2023). Refining the evolutionary tree of the horse Y chromosome. Sci. Rep..

[23] Stachurska A., Nogaj A., Brodacki A., Nogaj J., Batkowska J. (2014). Genetic distances between horse breeds in Poland estimated according to blood protein polymorphism. Czech J. Anim. Sci..

[24] Opora J. (2006). Die Wildbahngestüte Westfalens. Geschichte, Entwicklung und Zukunft.

[25] Lovász L., Fages A., Amrhein V. (2021). Konik, Tarpan, European wild horse: An origin story with conservation implications. Glob. Ecol. Conserv..

[26] Gmel A.I., Mikko S., Ricard A., Velie B.D., Gerber V., Hamilton N.A., Neuditschko M. (2024). Using high-density SNP data to unravel the origin of the Franches-Montagnes horse breed. Genet. Sel. Evol..

[27] Reke A., Zarina A., Vinogradovs I. (2019). Management of semi-wild large herbivores’ grazing sites in Latvia. Proceedings of the 12th International Scientific and Practical Conference.

[28] Vetulani T. (1928). Dalsze Badania nad Konikiem Polskim (T.67). Doctoral dissertation.

