# Supplementary material for: Whole Genome Data Uncover the Complex Origins of Polish Konik Horses
Source: Animals (Basel). 2026 May 29;16(11):1669. doi: 10.3390/ani16111669 (PMC13255651; doi:10.3390/ani16111669)
Supplement: Supplementary file 1 [file animals-16-01669-s001.zip › Data S2.pdf]

1	pc1	pc2	group
2	-0.267187	-0.0440079	sp4
3	-0.264272	-0.0486412	sp3
4	-0.264035	-0.0517489	sp2
5	-0.26417	-0.0505513	sp1
6	-0.176419	-0.129946	SRR2142313
7	-0.388524	-0.119023	SRR1564427
8	-0.215517	-0.0498057	SRR1048526
9	-0.23293	0.0623327	ERR11180374
10	-0.185724	-0.0648515	ERR6798762
11	-0.209973	-0.0832894	ERR6798756
12	-0.174124	-0.0738495	ERR6466048
13	-0.17575	-0.0641773	ERR6465818
14	-0.335901	-0.0899304	ERR982714
15	-0.179038	-0.0646393	ERR6465812
16	-0.177786	-0.0557823	ERR6465807
17	-0.00304946	0.0885135	BG
18	0.0324719	-0.0462007	AR
19	0.0326168	-0.0370245	AR
20	0.0318578	-0.0362476	AR
21	0.0384598	-0.0389111	AR

22	0.0317057	-0.0404102	AR
23	0.0449491	-0.0515458	AR
24	0.0184142	0.0199185	CP
25	0.0118165	0.0235205	CP
26	0.0155285	0.0162752	CP
27	0.0167206	0.0118237	CP
28	-0.00325043	0.109478	Halfinger
29	0.00112141	0.0737023	Franches
30	0.00233868	0.0788238	Franches
31	0.00280266	0.0731294	Franches
32	-0.00105025	0.0739408	Franches
33	0.00985277	0.0409062	Franches
34	0.00613651	0.0712872	Franches
35	0.00348407	0.0600804	Franches
36	0.00502374	0.0754725	Franches
37	-0.00191884	0.0816777	Franches
38	0.00844146	0.0503911	Franches
39	0.00204095	0.0697655	Franches
40	0.0360157	-0.0322115	FT
41	0.0399065	-0.0405498	FT
42	0.0408369	-0.034116	FT

43	0.0375623	-0.0239553	FT
44	0.0416119	-0.0531447	FT
45	0.0364274	-0.035788	FT
46	0.0379425	-0.0291562	FT
47	0.0377258	-0.0358525	FT
48	0.0375313	-0.0400378	FT
49	0.037936	-0.0401131	FT
50	-0.00696176	0.116221	Halfinger
51	-0.0127696	0.108814	Halfinger
52	-0.0106069	0.100248	Halfinger
53	-0.00789084	0.0938433	Halfinger
54	-0.00870853	0.113433	Halfinger
55	-0.010534	0.103583	Halfinger
56	-0.0133986	0.105624	Halfinger
57	-0.019472	0.0639862	Icelandic
58	-0.0315053	0.0864297	Icelandic
59	0.0376261	-0.0282579	SB
60	0.0379723	-0.0287961	SB
61	0.0401682	-0.0249042	SB
62	0.0362867	-0.0213588	SB
63	0.0361828	-0.0238723	SB

64	0.0295994	-0.0190404	SB
65	0.00931283	0.01063	MORG
66	0.011203	0.0135876	MORG
67	0.0404714	-0.0294063	SB
68	0.041418	-0.0284074	SB
69	-0.0129692	0.113402	BG
70	0.00544677	0.144254	Clyd
71	0.00206133	0.14138	Clyd
72	0.00281493	0.144788	Clyd
73	0.00732048	0.109921	Clyd
74	-0.000294292	0.147814	Clyd
75	0.00136461	0.145782	Clyd
76	0.0072011	0.10154	Clyd
77	0.0155505	-0.0412579	Clyd
78	0.00523496	0.130485	Clyd
79	-0.00867214	0.107549	BG
80	-0.0106289	0.106266	BG
81	-0.00821811	0.10793	BG
82	-0.00902689	0.1103 BG	
83	-0.00732959	0.108895	BG
84	-0.00805743	0.112177	BG

85	-0.00711423	0.113556	BG
86	-0.0101167	0.116045	BG
87	-0.0252344	0.0836158	Icelandic
88	-0.0248515	0.0890864	Icelandic
89	-0.0231256	0.0887383	Icelandic
90	-0.017766	0.0784161	Icelandic
91	-0.019414	0.0763573	Icelandic
92	-0.0218973	0.0807663	Icelandic
93	-0.0225977	0.0811023	Icelandic
94	0.0329839	-0.0248934	SB
95	0.0362066	-0.0302606	SB
96	0.0228676	-0.0135863	MORG
97	0.0158329	0.00616498	WP
98	0.0123088	0.014448	MORG
99	0.0214243	-0.0138743	MORG
100	0.0223766	-0.0181154	MORG
101	0.0180601	0.00775171	MORG
102	0.0191318	-0.00541995	MORG
103	0.0244368	-0.0116502	MORG
104	0.0216223	-0.000715216	MORG
105	0.00662867	0.0284583	WP

106	0.00887886	0.0210519	WP
107	0.0136641	-0.000749537	WP
108	0.000766083	0.0338426	WP
109	0.00294429	0.0279972	WP
110	0.0237434	-0.0225588	WP
111	0.00925334	0.0149181	WP
112	-0.00706559	0.0565258	WP
113	0.0155056	-0.128658	JP
114	-0.0334654	0.0434746	JP
115	0.0160091	-0.0783775	JP
116	0.0163241	-0.0951496	JP
117	0.0161029	-0.0863014	JP
118	0.016491	-0.0759431	JP
119	0.0170181	-0.0795842	JP
120	0.0172337	-0.0884983	JP
121	0.0148754	-0.0679424	JP
122	0.0545676	-0.131922	TH
123	0.0581131	-0.142191	TH
124	0.0604934	-0.143387	TH
125	0.0614908	-0.145521	TH
126	0.0610277	-0.141434	TH

127	0.0631665	-0.142745	TH
128	0.0589371	-0.142819	TH
129	0.0599559	-0.14373	TH
130	0.0275109	-0.0480363	WB
131	0.0614919	-0.142997	TH
132	0.0600924	-0.142827	TH
133	0.0417626	-0.0744743	WB
134	0.0545949	-0.136757	TH
135	0.00623471	0.0277002	CP
136	0.00637096	0.0179422	CP
137	-0.00548849	0.0567806	CP
138	-0.000602203	0.0408121	CP
139	0.0571888	-0.14324	TH
140	0.059442	-0.135478	TH
141	0.0364104	-0.0796811	WB
142	0.0459738	-0.0647859	AR
143	0.0449814	-0.0673601	AR
144	0.0404411	-0.0712591	WB
145	0.0470041	-0.0703904	AR
146	0.0502694	-0.0768205	AR
147	0.0423179	-0.059778	AR

148	0.0298004	-0.0631921	WB
149	0.0367329	-0.0714692	WB
150	0.0284272	-0.0517906	WB
151	-0.0240614	0.1181	WB
152	0.0333877	-0.0664897	WB
153	0.0347675	-0.071434	WB
