# Supplementary material for: Whole Genome Data Uncover the Complex Origins of Polish Konik Horses
Source: Animals (Basel). 2026 May 29;16(11):1669. doi: 10.3390/ani16111669 (PMC13255651; doi:10.3390/ani16111669)
Supplement: Supplementary file 1 [file animals-16-01669-s001.zip › Table S1.pdf]

Sample	Raw reads	Filtered reads	Mapped reads (quality above 30)	Percent of mapping
KP1	82968284	81341455	72856728	89.56
KP2	83891928	82246988	74527591	90.61
KP3	83269253	81636523	73320681	89.81
KP4	83200692	81569306	74039316	90.76
