# Supplementary figures and images for: Whole Genome Data Uncover the Complex Origins of Polish Konik Horses
Source: Animals (Basel). 2026 May 29;16(11):1669. doi: 10.3390/ani16111669 (PMC13255651; doi:10.3390/ani16111669)

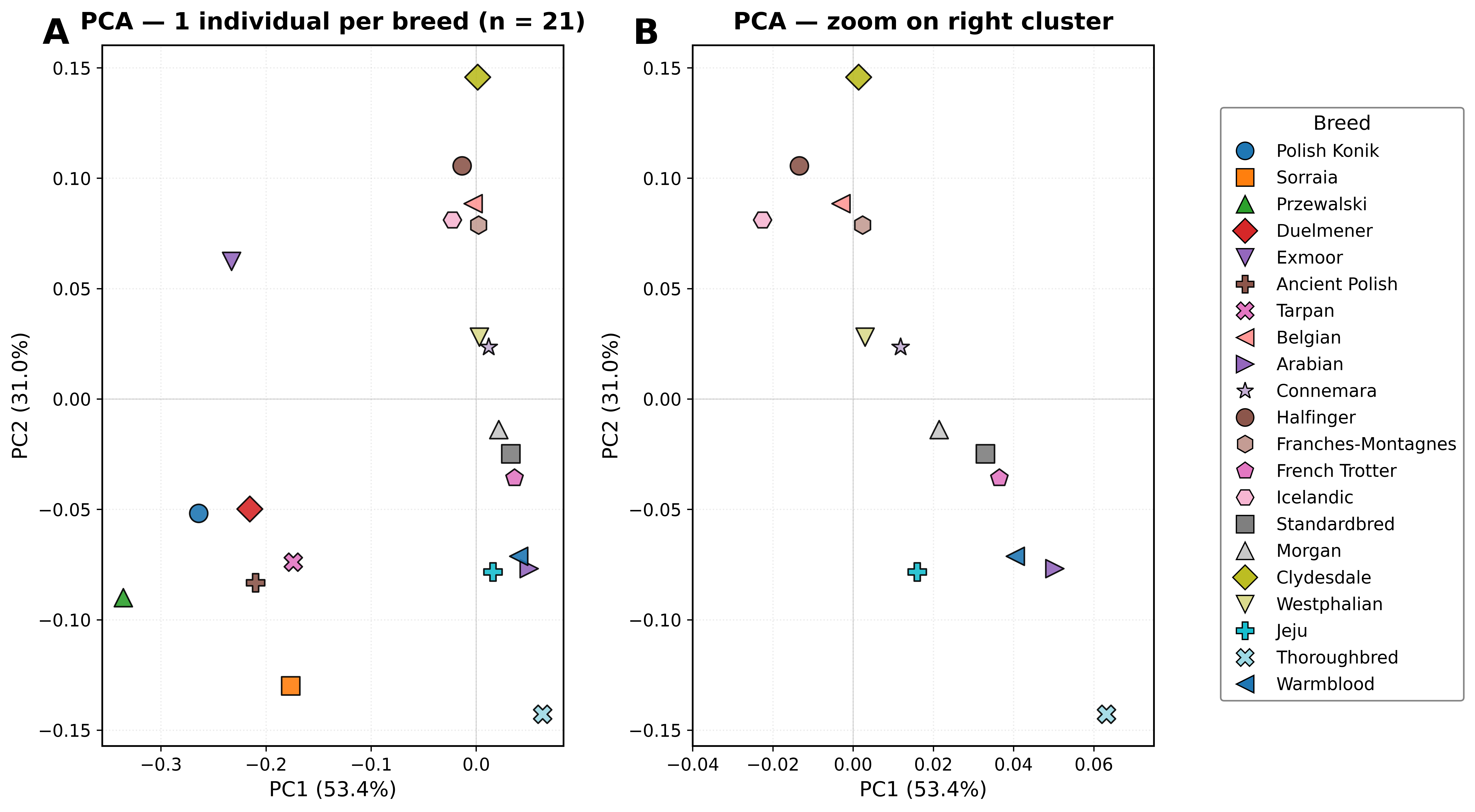

Supplement: Supplementary file 1 [file animals-16-01669-s001.zip › Figure S1.png]

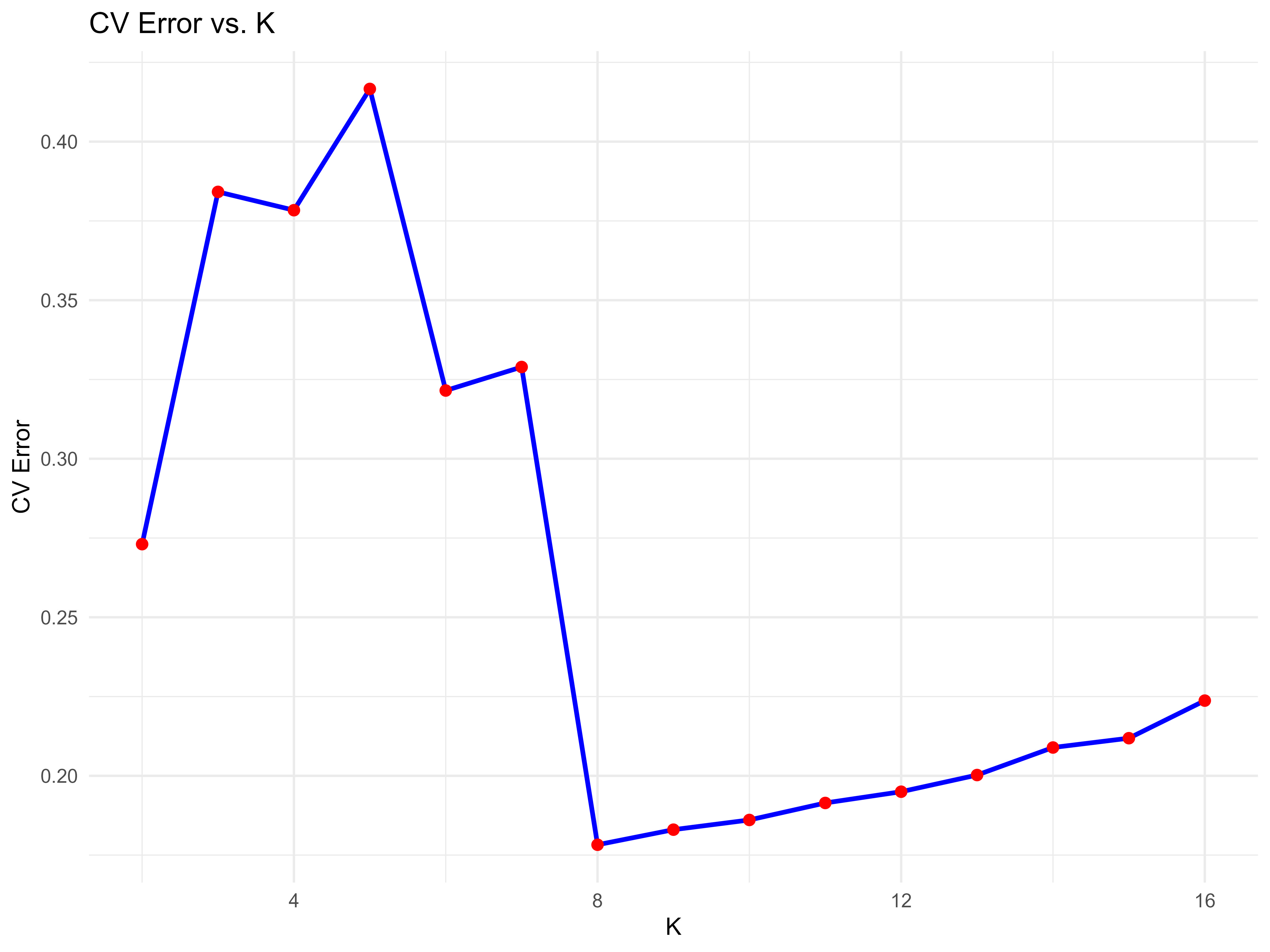

Supplement: Supplementary file 1 [file animals-16-01669-s001.zip › Figure S2.png]

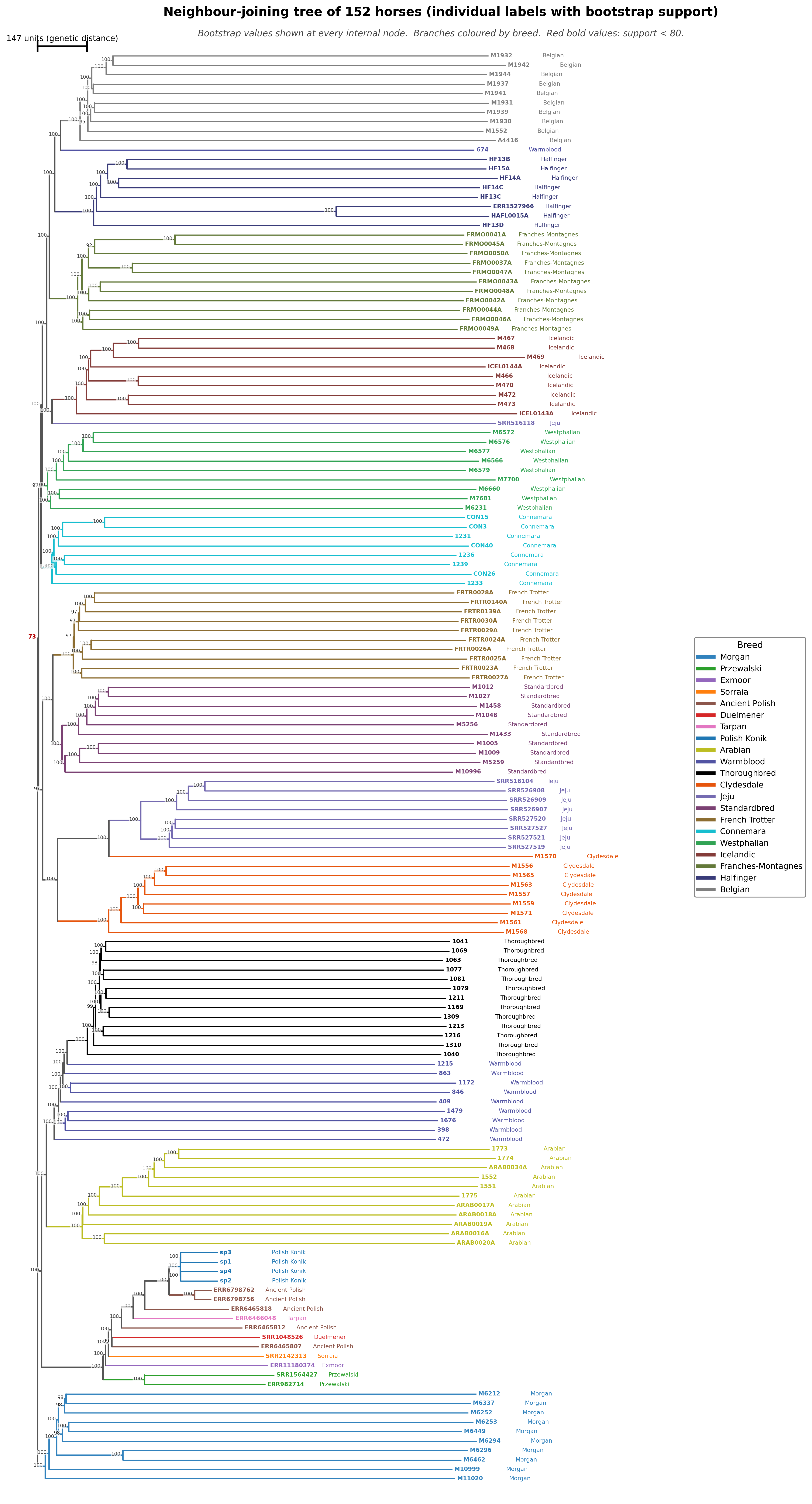

Supplement: Supplementary file 1 [file animals-16-01669-s001.zip › Figure S4.png]
